# Editorial: Point-of-care testing for infectious and foodborne pathogens, volume II

**DOI:** 10.3389/fcimb.2023.1219506

**Published:** 2023-06-26

**Authors:** Hongchao Gou, Jianmin Zhang, Ming Liao

**Affiliations:** ^1^ Institute of Animal Health, Guangdong Academy of Agricultural Sciences, Guangdong Provincial Key Laboratory of Livestock Disease Prevention, Key Laboratory for Prevention and Control of Avian Influenza and Other Major Poultry Diseases, Maoming Branch, Guangdong Laboratory for Lingnan Modern Agriculture, Scientific Observation and Experiment Station of Veterinary Drugs and Diagnostic Techniques of Guangdong Province, Guangzhou, China; ^2^ National and Regional Joint Engineering Laboratory for Medicament of Zoonoses Prevention and Control, Key Laboratory of Zoonoses, Ministry of Agriculture, Key Laboratory of Zoonoses Prevention and Control of Guangdong Province, Key Laboratory of Animal Vaccine Development, Ministry of Agriculture, Guangdong Laboratory for Lingnan Modern Agriculture, College of Veterinary Medicine, South China Agricultural University, Guangzhou, China

**Keywords:** infectious and foodborne pathogens, point-of-care testing (POCT), Isothermal amplification, recombinase polymerase amplification (RPA), loop-mediated isothermal amplification (LAMP)

Infectious and foodborne pathogens usually pose emergency threat to the human and animal health. In recent years, they are gaining more attention due to emerging and re-emerging outbreaks, such as Influenza virus, Severe acute respiratory syndrome virus (SARS), Severe acute respiratory syndrome coronavirus 2 (SARS-CoV-2), Ebola virus, Norovirus, *Salmonella Typhimurium*, *Escherichia coli* O157, Hepatitis E Virus, African swine fever virus and so on. Rapid and early detection of infectious and foodborne pathogens display a dramatic impact in controlling and preventing an outbreak. To face current challenges regarding infectious and foodborne pathogens, a point-of-care testing (POCT) concept has been introduced to detection technologies and devices. POCT technologies displayed the advance of user-friendly, rapid detection, so they can be directly used on-site or at home. This Research Topic is focused on novel ideas in POCT technologies and devices for infectious and foodborne pathogens, aimed at improving on-site application of rapid diagnostic techniques by detecting analytes including antigens, nucleic acids and specific antibodies for microorganisms.

Influenza A viruses (IAVs) are important pathogens of respiratory infections, causing not only seasonal influenza but also influenza pandemics and posing a global threat to public health. IAVs infection spreads rapidly, widely, and across species, causing huge losses, especially zoonotic IAVs infections that are more harmful. In this Research Topic, Cui et al. established a real-time reverse transcription recombinase-aided amplification (real-time RT-RAA) assay targeting conserved positions in the matrix protein gene (M gene) of IAVs. The assay can be completed within 20 min at 42°C. The sensitivity of the real-time RT-RAA assay was 142 copies per reaction at 95% probability, which was comparable to the sensitivity of the RT-qPCR assay. The specificity assay showed that the real-time RT-RAA assay was specific to IAVs, and there was no cross-reactivity with other important viruses. In addition, 100% concordance between the real-time RT-RAA and RT-qPCR assays was achieved after testing 65 clinical specimens. The results suggested that the real-time RT-RAA assay was a specific, sensitive and reliable diagnostic tool for the rapid detection of IAVs. It will be critical for fast and sensitive detection of IAVs and controlling the spread of this disease.

Hepatitis E virus (HEV) is a zoonotic pathogen that causes global hepatitis E. Outbreaks of Hepatitis E are directly linked to the consumption of pork liver products. Herein, Wang et al. developed a reverse transcription recombinase polymerase amplification assay targeting the ORF2 gene to rapid detect HEV by integrating the fluorescence detection platform (qRT-RPA) and the visible lateral flow biosensor by naked eyes (LFB RT-RPA). The qRT-RPA assay effectively detected HEV RNA with a limit of detection (LOD) of 154 copies/μL at 41°C for 20 min. Besides, the LFB RT-RPA detected the HEV RNA with a LOD of 24 copies/μL. The developed RT-RPA assays also showed good specificity for HEV with no cross-reactions with any of the other important swine pathogens examined in this work. The performance of the developed RT-RPA assays was validated on 14 HEV RNA positive and 66 HEV RNA negative raw pork liver samples identified by a previously described qRT-PCR. Consequently, 11 and 12 samples were HEV RNA positive as detected by the qRT-RPA and LFB RT-RPA, respectively. Compared to qRT-PCR, the qRT-RPA and LFB RT- RPA assays revealed a coincidence rate of 96.3% and 97.5%, as well as a Kappa value of 0.858 and 0.908, respectively. These results ascertain that the developed RT-RPA assays are effective diagnostic tools for the point-of-care detection of HEV in resource-limited settings.

Sexually transmitted chlamydia and gonorrhea infections caused by the bacteria *Chlamydia trachomatis* and *Neisseria gonorrhoeae* remain a major public health concern worldwide, particularly in less developed nations. It is crucial to use a POCT diagnostic method that is quick, specific, sensitive, and user-friendly to treat and control these infections effectively. Here, Chen et al. devised a novel molecular diagnostic assay, combining multiplex loop-mediated isothermal amplification (mLAMP) with a visual gold nanoparticles-based lateral flow biosensor (AuNPs-LFB), used for highly specific, sensitive, rapid, visual, and easy identification of *Chlamydia trachomatis* and *Neisseria gonorrhoeae*. Two unique independent primer pairs were successful designed against the ompA and orf1 genes of *C. trachomatis* and *N. gonorrhoeae*, respectively. The optimal mLAMP-AuNPs-LFB reaction conditions were determined to be 67°C for 35 min. The detection procedure, involving crude genomic DNA extraction (~5 min), LAMP amplification (35 min), and visual results interpretation (<2 min), can be completed within 45 min. This assay has a detection limit of 50 copies per test, displaying no cross-reactivity with any other bacteria. Hence, this assay can potentially be used for POCT to detect *C. trachomatis* and *N. gonorrhoeae* in clinical settings, particularly in underdeveloped regions.

As to *Streptococcus suis* infection, serotype 2 and 14 is the most prevalent zoonotic strains. The establishment of a sensitive and extremely accurate method for POCT of *Streptococcus suis* serotype 2 and 14 strains on-site is highly desired. A loop primer probe-introduced loop-mediated isothermal amplification assay was developed by Meng et al. to differentiate *Streptococcus suis serotype* 2 and 14 based on SNP. The specific fluorescent probes were designed for the SNP site specific for serotype 2 and 14 *Streptococcus suis* cpsK genes, and the loop primer probe-introduced loop-mediated isothermal amplification assay was activated by the specific cleavage properties of RNase H2 enzyme. By using swarm primers to accelerated speed and efficiency of LAMP assays, the amplification reaction can be performed efficiently at isothermal 59°C within 40 minutes. The resuts can be real time detected or judged by using a smartphone and a 3D printed visualization cassette. The sensitivity of the *Streptococcus suis* LAMP assay can reach 18.4 CFU. The detection rate of the assay system was evaluated using clinical 19 samples with suspected *Streptococcus suis* infection, and the detection rate was consistent with the sequencing method, which is highly practical. This assay is specific and sensitive, not dependent on instruments and professional operators. Therefore, it is potential for field testing and differentiation of *Streptococcus suis* serotypes 2 and 14.

Genotype II African swine fever virus (ASFV) has been plaguing Asian pig industry since 2018. Recently, genotype I ASFV was reported for the first time in China. Since there is no commercial vaccine available against ASFV, early onsite detection and quick culling procedures are commonly used by many countries all over the world. It is desirable that the above two genotypes of ASFV could be quickly differentiated during onsite detection at the same time. Song et al. reported a sensitive and simple Fluorescent Probe Hydrolysis-Insulated isothermal PCR (iiPCR) that can detect and differentiate two genotypes of ASFV within 40 minutes. The positive or negative results of tested samples displayed on the screen of device automatically after PCR amplification was complete. The detection limit of the iiPCR was tested to be 20 copies for both genotype I and genotype II ASFVs. There was no cross-reactivity with other swine viruses by using the established iiPCR. Fifty-eight ASFV positive samples confirmed by National ASF Reference Laboratory were subjected to the established duplex iiPCR for genotype differentiation. The results showed that all these ASFV-positive samples belong to genotype II. Meanwhile, they found serum samples could be directly used as the templates for iiPCR without comprising sensitivity and specificity. Therefore, the duplex iiPCR was proved to be a useful tool for ASFV onsite detection and genotype differentiation.

In general, RPA, LAMP and isothermal PCR methods were mainly employed for POCT of infectious and foodborne pathogens in This Research Topic. Compared with RPA, LAMP and isothermal PCR methods only utilize single enzyme to realize the amplification reaction, showing huge advantage in use-cost. However, most reports in this Research Topic have not yet to be fully applied in POCT. Complex nucleic acid extraction process is the critical defectives of these methods. Indeed, POCT requires that all of the analytical processes, from sample collection to result communication, should be performable in one or a few simple steps to reduce time and costs. In addition to new amplification methods and devices, rapid nucleic acid extraction methods suitable for POCT should also be paid more attention in the future.

## Author contributions

HG drafted the manuscript. JZ and ML participated the revision of this manuscript. All the authors read and approved the final manuscript.

